# Differential diagnosis of LTP-sensitised patients in Central Europe

**DOI:** 10.1186/2045-7022-5-S3-P138

**Published:** 2015-03-30

**Authors:** Nadine Mothes-Luksch, Marianne Raith, Georg Stingl, Stefan Wohrl, Ines Swoboda

**Affiliations:** 1 Comparative Immunology and Oncology, Department of Pathophysiology and Allergy Research, Center of Pathophysiology, Infectiology and Immunology, Medical University of Vienna, Vienna, Austria; 2 Molecular Biotechnology Section, University of Applied Sciences, FH Campus Wien, Campus Vienna Biocenter, Vienna, Austria; 3 Department of Dermatology, Division of Immunology, Allergy and Infectious Diseases, Medical University of Vienna, Vienna, Austria; 4 FAZ – Floridsdorf Allergy Center, Vienna, Austria

## 

Lipid transfer protein (LTP) is a major allergen in peach often causing anaphylactic reactions with a high prevalence of sensitization in the Mediterranean area. The prevalence of LTP sensitization in Central Europe is currently unknown, where the diagnosis is complicated by a high background sensitization to the major birch pollen allergen, Bet v 1, and Bet v 1 cross-reactive food allergens, which cause only mild reactions. LTP and Bet v 1-related proteins are both distributed throughout the plant-kingdom. In this study, we aimed to characterize the sensitization-patterns of patients with severe reactions to plant-derived food in Austria.

Thirteen patients reporting strong reactions after consumption of fruits, vegetables or herbs were invited to skin prick and prick to prick tests. For visualizing patients' IgE-reactivity patterns, immunoblots were performed, which allowed the determination of specific IgE to raw and boiled parsley, raspberry, apricot, and peach. These immunoblots showed that cooking did not influence IgE binding to LTPs, while cooking reduced IgE binding to all Bet v 1-related proteins. We further observed that LTP-sensitized patients showed variable IgE reactivity to different LTPs, however, the strongest reactions were seen to fruits from the Rosaceae family, especially to peach, suggesting that peach LTP (Pru p 3) contains the majority of IgE epitopes also for a Central European population. Analysis of IgE reactivity to single molecules (including Bet v 1 and Pru p 3) using an allergen micro-array (ISAC) and single ImmunoCAP measurements enabled to determine the following sensitization patterns: 1 patient was monosensitized to Bet v 1, 3 were monosensitized to LTP, whereas 8 showed a co-sensitization to Bet v 1 and LTP and 1 patient was monosensitized to profilin.

Based on this study, we conclude that analysis of specific IgE to the LTP Pru p 3 from peach is also sufficient for diagnosis of reactions to Rosacea fruits even in regions where frequent co-sensitization to Bet v 1 makes the interpretation of extracts-based skin prick tests difficult. In addition, also other plant-related panallergens, such as profilin, should be included in such a differential diagnostic workup scheme.

